# miR-20b Inhibits T Cell Proliferation and Activation via NFAT Signaling Pathway in Thymoma-Associated Myasthenia Gravis

**DOI:** 10.1155/2016/9595718

**Published:** 2016-10-19

**Authors:** Yanzhong Xin, Hongfei Cai, Tianyu Lu, Yan Zhang, Yue Yang, Youbin Cui

**Affiliations:** Department of Thoracic Surgery, The First Hospital of Jilin University, Changchun, Jilin 132000, China

## Abstract

*Purpose*. We examined the role of miR-20b in development of thymoma-associated myasthenia gravis, especially in T cell proliferation and activation.* Materials and Methods*. Using qRT-PCR, we assessed expression levels of miR-20b and its target genes in cultured cells and patient samples and examined the proliferation of cultured cells, using MTT cell proliferation assays and flow cytometry based cell cycle analysis. Activation of T cells was determined by both flow cytometry and qRT-PCR of activation-specific marker genes.* Results*. Expression of miR-20b was downregulated in samples of thymoma tissues and serum from patients with thymoma-associated myasthenia gravis. In addition, T cell proliferation and activation were inhibited by ectopic overexpression of miR-20b, which led to increased T cell proliferation and activation. NFAT5 and CAMTA1 were identified as targets of miR-20b. Expression levels of NFAT5 and CAMTA1 were inhibited by miR-20b expression in cultured cells, and the expression levels of miR-20b and NFAT5/CAMTA1 were inversely correlated in patients with thymoma-associated myasthenia gravis.* Conclusion*. miR-20b acts as a tumor suppressor in the development of thymoma and thymoma-associated myasthenia gravis. The tumor suppressive function of miR-20b in thymoma could be due to its inhibition of NFAT signaling by repression of NFAT5 and CAMTA1 expression.

## 1. Introduction

Thymoma is a thymic tumor initiated by neoplasia of epithelial cells. It is a rare tumor type associated with the paraneoplastic autoimmune disease, myasthenia gravis [1], which develops in about half of thymoma patients [2, 3]. Thymomas are unique in their ability to generate mature T cells from immature precursors [4]. To date, limited information is available about the biology of thymoma, in particular the deregulated pathways at the transcriptional and posttranscriptional levels.

Recently, there has been accumulating evidence that microRNAs act as either oncogenes or tumor suppressors in many tumor types [5, 6]. MicroRNAs (miRNAs) are 19–22-nucleotide RNAs that regulate gene expression, primarily, posttranscriptionally, by complementary binding to the 3′-UTR of target mRNA, resulting in mRNA destabilization and/or translational inhibition [7]. Interestingly, a number of fundamental biological processes essential for the onset and development of tumors, including cell proliferation [8], cell differentiation [9], and cell invasion [10], have all been reported to be subjected to the regulation of miRNAs.

The role of miR-20b in tumor development was recently characterized. In MCF-7 breast cancer cells, miR-20b was shown to target HIF-1 alpha and STAT3, thereby affecting VEGF expression [11]. miR-20b also regulates the expression of the proteinase-activated receptor-1 (PAR-1) thrombin receptor in melanoma cells [12]. It has been demonstrated to regulate several cancers, including gastric [13], liver [14], cervical [15], colon [16], lung [17], and lymphoma [18]. However miR-20b's function in thymoma development and implications of thymoma in myasthenia gravis pathophysiology have not been previously systematically investigated.

In this study, we aim to analyze the expression levels to miR-20b in thymoma tissues and investigate the effect of miR-20b in T cell development and then discover the potential molecular mechanisms.

## 2. Materials and Methods

### 2.1. Human Samples

Thymoma tissue specimens and serum samples were obtained from myasthenia gravis patients at the First Affiliated Hospital of Jilin University and stored in liquid nitrogen. CD4^+^ cells were isolated from participant blood samples, using the Dynabeads® CD4 Positive Isolation Kit (Thermo Fisher Scientific, San Jose, CA, USA) according to manufacturer's instructions. Prior written informed consent was obtained from all the participants. The study was approved by the Institutional Ethics Board of the First Affiliated Hospital of Jilin University.

### 2.2. Cell Culture and T Cell Activation

Jurkat T cells and human embryo kidney cell (HEK293T) were maintained in Dulbecco's minimum essential medium (DMEM, Gibco BRL, Grand Island, NY, USA) with 10% fetal bovine serum (FBS, Invitrogen, Carlsbad, California, USA) and 1% penicillin/streptomycin. All cells were cultured in an incubator with 5% CO_2_ at 37°C. Lipofectamine 2000 (Invitrogen) was used for miRNA transfection. Cells were assayed 48 h after transfection. Jurkat and human peripheral blood CD4^+^ T cells were activated, using anti-CD3 and anti-CD28 monoclonal antibodies (BD Biosciences, San Jose, CA, USA), as described previously [19].

### 2.3. Quantitative Real-Time PCR (qRT-PCR)

TRIZol reagent (Invitrogen) was used to extract total RNA. Using a QuantiTect Reverse Transcription kit (Qiagen, Valencia, CA, USA), 2 *μ*g of each RNA was reverse-transcribed into cDNA. Expression levels of the genes were analyzed using a QuantiTect SYBR Green PCR kit (Qiagen). qRT-PCR was then performed on an ABI Prism 7900 fast RT-PCR system (Applied Biosystems, Foster City, CA, USA). The primer sequences were listed as below:

 NFAT5: forward: 5′-CACCGCTTGTCTGACTCATT-3′, reverse: 5′-GCCCAAGTCCCTCTACTCG-3′.
 CAMTA1: forward: 5′-AGTGCAGAAAATGAAGAATGCG-3′, reverse: 5′-CAAAATTCTCCTGCTTGATTCG-3′.
 GAPDH: forward: 5′-GTCATCATCTCCGCCCCTTCTGC-3′, reverse: 5′-GATGCCTGCTTCACCACCTTCTTG-3′.
 IL-2: forward: 5′-CAGGATGGAGAATTACAGGAACCT-3′,
 reverse: 5′-TTTCAATTCTGTGGCCTGCTT-3′.
 IL-10: forward: 5′-AAGCTGAGAACCAAGACCCAGACATCAAGGCG-3′,
 reverse: 5′-AGCTATCCCAGAGCCCCAGATCCGATTTTGG-3′.

### 2.4. MTT Assay

Jurkat cells (4 × 10^3^/well cells) were transfected with miRNA and then seeded into 96-well plates in 100 *μ*L of medium for 48 h. After an additional 0, 1, 2, 3, or 4 days, 10 *μ*L (10 *μ*g/mL) 3-(4,5-dimethylthiazol-2-yl)-2,5-diphenyltetrazolium bromide (MTT; Sigma, St. Louis, MO, USA) was added to each well, and cells were incubated for 4 h at 37°C. Supernatant was removed, and 100 *μ*L DMSO was added to dissolve the formazan production. Jurkat cell proliferation was assayed by measuring absorbance at 490 nm (OD values).

### 2.5. Cell Cycle Analysis

Jurkat cells were harvested by trypsinization, collected, and washed three times with cold PBS and fixed overnight in ice-cold 70% ethanol. After centrifugation, supernatant was discarded and the cell pellets stained with 80 *μ*g/mL of propidium iodide and 100 *μ*g/mL of RNase A and were then incubated in PBS for 30 min at room temperature in the dark.

DNA content was evaluated by a flow cytometry (BD Biosciences) with FlowJo software to calculate percentages of cells in different phases of the cell cycle; DNA contents were analyzed.

### 2.6. Immunostaining and Flow Cytometric Analysis

Jurkat cells were stained with fluorescein-labeled monoclonal antibodies specific for CD25 and CD69 (BD Biosciences). The percentages of CD25^+^ and CD69^+^ cells were determined by flow cytometry (BD Biosciences) as previously described [20].

### 2.7. Luciferase Assay

3′-UTR luciferase reporter assays were performed in human HEK293T cells. The 3′-UTR of NFAT5 and CAMTA1 with potential miR-20b binding sites was cloned from the genome of Jurkat cells and then placed into pmiR-REPORT vectors (Promega, Madison, WI, USA). HEK293T cells were cotransfected with the constructed luciferase reporter plasmids and miRNA. Renilla luciferase plasmids were cotransfected and used for normalization of transfection efficiency. After 48 h of transfection, luciferase activity was measured using a Dual-Luciferase Reporter Assay System (Promega).

### 2.8. Western Blot

Cells were lysed with RIPA lysis buffer with protease inhibitor (Boster, Wuhan, Hubei, China). Lysates were centrifuged at 4°C, 12,000 ×g for 10 min, supernatants collected, and protein concentrations assessed using a BCA protein assay kit (Boster). Equal amounts of protein were placed on 10% SDS-PAGE gels and blotted onto polyvinylidene difluoride membranes (Bio-Rad, Hercules, CA, USA). Membranes were blocked with 5% nonfat milk for 1 h at room temperature and then probed with NFAT5, CAMTA1, and GAPDH antibodies (Santa Cruz, Santa Cruz, CA, USA) at 4°C overnight. The blots were then incubated with HRP-conjugated secondary antibody. GAPDH was used as an endogenous protein control. ECL substrates were used to visualize signals (Boster).

### 2.9. Statistics

The data are expressed as the mean ± SD of three independent experiments and processed by GraphPad Prism software (GraphPad Software Inc., San Diego, USA). Student's* t*-test, one-way ANOVA Spearman, and Pearson correlation analyses were used to distinguish differences between groups. A *P* value of <0.05 was considered statistically significant.

## 3. Results

### 3.1. Expression Levels of miR-20b Are Downregulated in Thymoma Tissues and Serum from Patients with Thymoma-Associated Myasthenia Gravis, as well as Activated T cells

**Figure 1:**
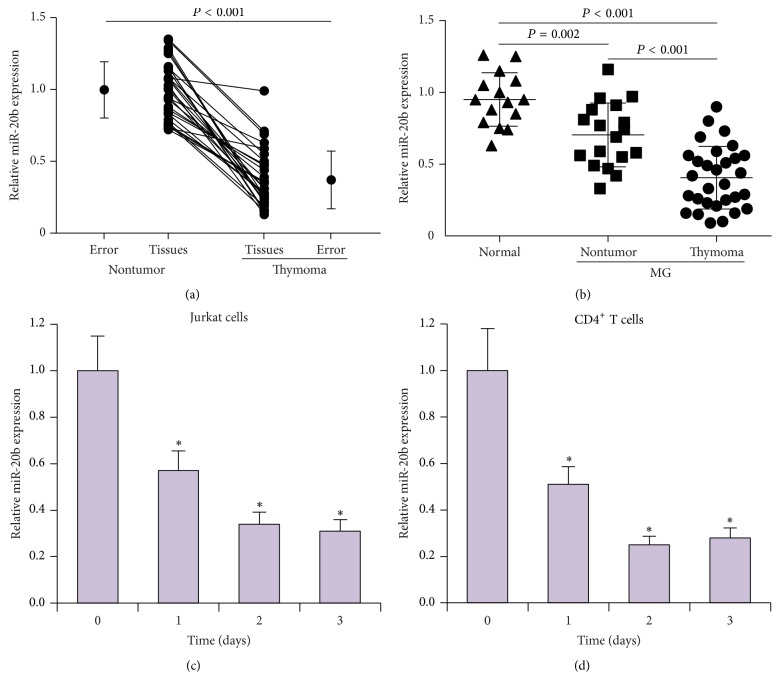
Expression levels of miR-20b are downregulated in thymoma tissue and serum samples from patients with thymoma-associated myasthenia gravis, as well as in activated T cell. (a) Expression levels of miR-20b as determined by qRT-PCR in thymoma tissue specimens from 30 thymoma-associated myasthenia gravis patients compared to those in adjacent nontumor tissue samples and (b) in serum samples from 30 patients with thymoma-associated myasthenia gravis, 18 patients without, and 15 normal samples from healthy participants. (c) Expression levels of miR-20b were determined by qRT-PCR at different time points (0, 1, 2, and 3, d) in activated Jurkat cells. (d) Human CD4^+^ cells were isolated from human peripheral blood, and miR-20b expression was determined by qRT-PCR at different time points (0, 1, 2, and 3, d) in activated CD4^+^ cells. ^*∗*^ *P* < 0.05.

It has been reported that the levels of miR-20b are downregulated in the serum of myasthenia gravis patients [21]. Since myasthenia gravis is the primary autoimmune manifestation of thymoma, we investigated the role of miR-20b during thymoma development. Expression levels of miR-20b were assessed in thymoma tissue specimens from 30 thymoma-associated myasthenia gravis patients, and we found that the levels of miR-20b were significantly decreased compared to those in adjacent nontumor tissues (Figure 1(a)). We examined the levels of miR-20b serum from myasthenia gravis patients with or without concurrent thymoma and found that levels of miR-20b were lower in myasthenia gravis patients compared to healthy participants. Interestingly, miR-20b levels were even lower in gravis patients with thymoma than those without thymoma (Figure 1(b)). To measure miR-20b expression in activated T cells, we activated Jurkat cells and human CD4^+^ T cells with anti-CD3 and anti-CD28 monoclonal antibodies and then examined miR-20b levels at 0, 1, 2, and 3 days. As shown in Figures 1(c) and 1(d), expression levels of miR-20b were downregulated in activated T cells, indicating that both the thymus and circulating levels of miR-20b were decreased in patients with thymoma-associated myasthenia gravis.

### 3.2. miR-20b Inhibits T Cell Proliferation and Activation

**Figure 2:**
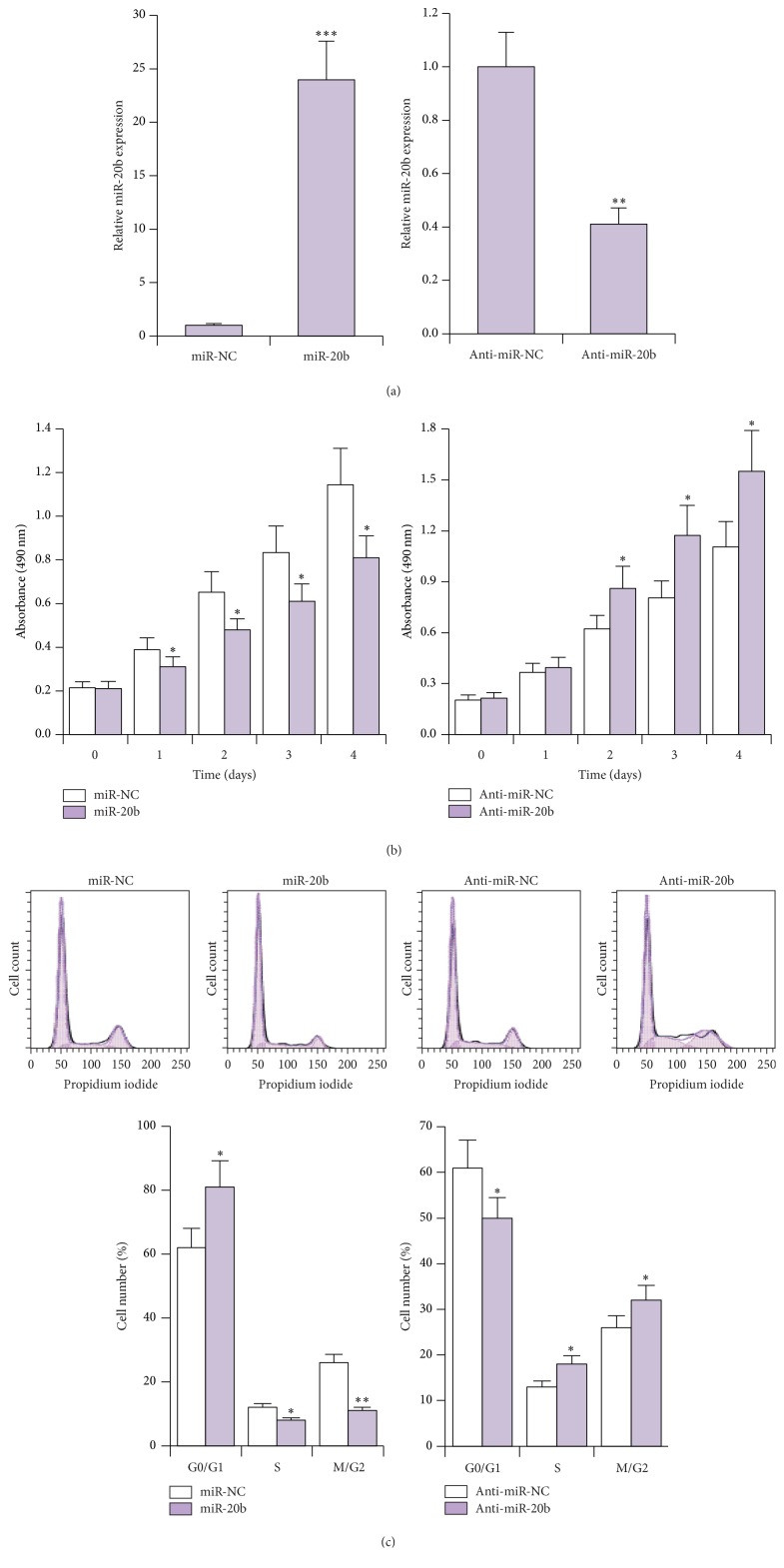
miR-20b inhibits T cell proliferation. (a) Expression levels of miR-20b as determined by qRT-PCR in Jurkat cells 48 h after transfection with either miR-20b or anti-miR-20b or their respective controls (miR-NC or anti-miR-NC). ^*∗∗*^ *P* < 0.01; ^*∗∗∗*^ *P* < 0.001. (b) Cell proliferation was determined by MTT assays in Jurkat cells transfected with either miR-20b or anti-miR-20b or their respective controls (miR-NC or anti-miR-NC). ^*∗*^ *P* < 0.05. (c) Flow cytometry based cell proliferation assays and quantification in Jurkat cells transfected with either miR-20b or anti-miR-20b or their respective controls (miR-NC or anti-miR-NC). ^*∗*^ *P* < 0.05; ^*∗∗*^ *P* < 0.01.

The ability of thymoma to cause myasthenia gravis has been shown to be dependent on its induction of the proliferation, maturation, and export of T cells [22]. To explore the role of miR-20b in T cell proliferation, we introduced miR-20b or miR-20b inhibitor (anti-miR-20b) into Jurkat cells and confirmed the efficacy of treatment (Figure 2(a)). We then performed MTT assay on these cells. We observed that miR-20b significantly inhibited and anti-miR-20b significantly promoted proliferation of Jurkat cells as compared to their respective negative controls (miR-NC and anti-miR-NC) (Figure 2(b)). These results were further corroborated by cell cycle analysis. We found that increased miR-20b expression induced G0/G1 arrest and that decreased miR-20b expression relieved G0/G1 arrest and promoted cell cycle progression (Figure 2(c)).

**Figure 3:**
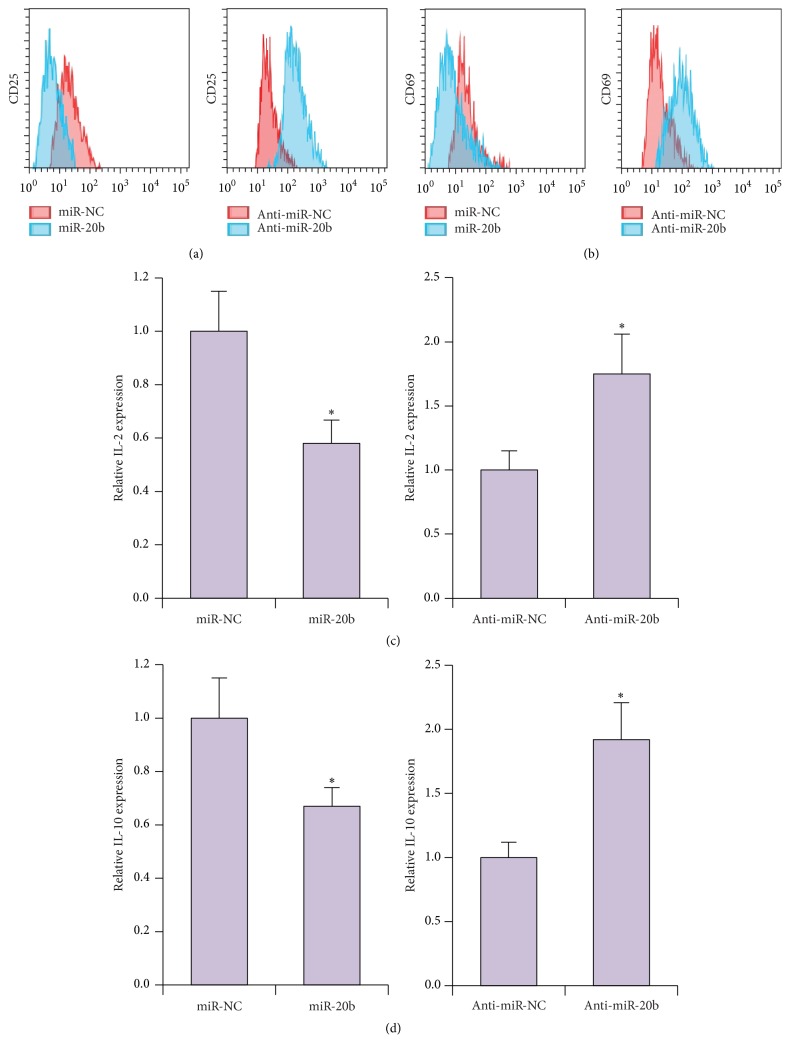
miR-20b inhibits T cell activation. (a) Flow cytometric analysis of CD25 and (b) CD69 expression in Jurkat cells transfected with either miR-20b or anti-miR-20b or their respective controls (miR-NC or anti-miR-NC). (c) Expression levels of IL-2 and (d) IL-10 as determined by qRT-PCR in Jurkat cells transfected with either miR-20b or anti-miR-20b or their respective controls (miR-NC or anti-miR-NC). ^*∗*^ *P* < 0.05.

We next determined the effects of miR-20b on T cell activation. To this end, we analyzed T cell CD25 and CD69 expression by flow cytometry. We found that increased miR-20b expression led to lower levels of both CD25 and CD69 (Figure 3(a)). In contrast, decreased miR-20b expression induced by anti-miR-20b treatment led to higher CD25 and CD69 levels (Figure 3(b)). Moreover, expression of IL-2 and IL-10 was lower in miR-20b-treated cells and higher in anti-miR-20b-treated cells (Figures 3(c) and 3(d)). These results indicated that increased expression of miR-20b inhibited proliferation and activation of T cells.

### 3.3. NFAT5 and CAMTA1 Are Targets of miR-20b

**Figure 4:**
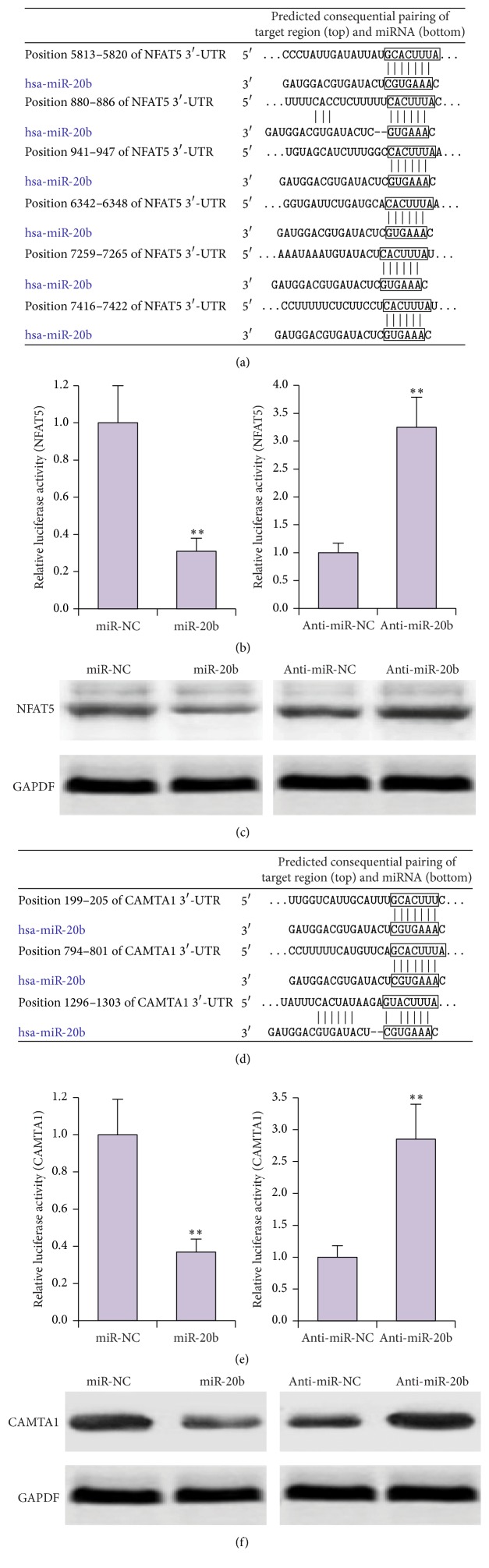
NFAT5 and CAMTA1 are targets of miR-20b. (a) Schematic diagram of putative binding sites of miR-20b in the 3′-UTR of NFAT5. (b) HEK293T cells were cotransfected with miR-20b and NFAT5 luciferase reporter vector. Luciferase activity was analyzed 48 h after transfection. (c) Protein levels of NFAT5 as determined by Western blot analysis in Jurkat cells transfected with miR-20b, anti-miR-20b, or their respective controls (miR-NC or anti-miR-NC). (d) Schematic diagram of putative binding sites of miR-20b in the 3′-UTR of CAMTA1. (e) HEK293T cells were cotransfected with miR-20b and CAMTA1 luciferase reporter vector. Luciferase activity was analyzed 48 h after transfection. (f) Protein levels of CAMTA1 as determined by Western blot analysis in Jurkat cells transfected with miR-20b, anti-miR-20b, or their respective controls (miR-NC or anti-miR-NC). ^*∗∗*^ *P* < 0.01.

To explore the mechanism of action of miR-20b in regulating T cell proliferation and activation, we identified potential targets with two target prediction algorithms, TargetScan (http://www.targetscan.org/) and miRanda (http://www.microrna.org/). Among the predicted miR-20b target genes, we were particularly interested in NFAT5 and CAMTA1, both of which are involved in regulating T cell proliferation [19, 23, 24]. We identified multiple sequences in the 3′-UTR of NFAT5 gene that were complementary to the miR-20b sequence (Figure 4(a)). To confirm our prediction, we generated a NFAT5 luciferase reporter plasmid and cotransfected the plasmid with miR-20b or anti-miR-20b into HEK293T cells. Luciferase activity was inhibited by miR-20b and promoted by anti-miR-20b transfection (Figure 4(b)). Overexpression of miR-20b in Jurkat cells decreased the levels of NFAT5 protein, whereas anti-miR-20b treatment increased it (Figure 4(c)).

Similarly, complementarity was also detected between the 3′-UTR of CAMTA1 gene and miR-20b (Figure 4(d)). Luciferase activity driven by CAMTA1 was inhibited by miR-20b and promoted by anti-miR-20b transfection (Figure 4(e)). In Jurkat cells, CAMTA1 protein levels were inhibited by miR-20b overexpression and enhanced by anti-miR-20b treatment (Figure 4(f)).

### 3.4. Expression Levels of miR-20b and NFAT5/CAMTA1 Were Inversely Correlated for Thymoma-Associated Myasthenia Gravis Patients

**Figure 5:**
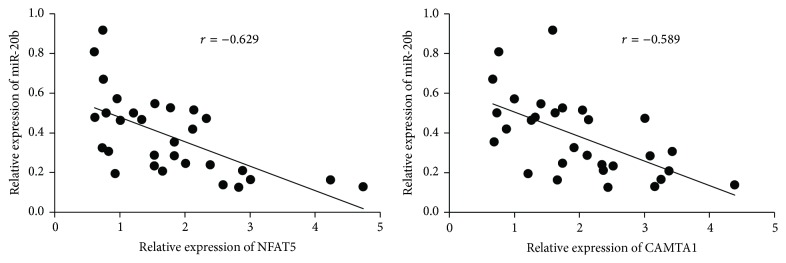
Expression levels of miR-20b and NFAT5/CAMTA1 were inversely correlated in thymoma-associated myasthenia gravis patients. Significant inverse correlation as determined by Spearman's correlation test was detected between the expression levels of miR-20b and those of NFAT5 and between the expression levels of miR-20b and those of CAMTA1, in samples from 30 patients with thymoma-associated myasthenia gravis.

To determine the physiological significance of miR-20b targeting NFAT5 and CAMTA1, we assessed the expression levels of miR-20b, NFAT5, and CAMTA1 in thymoma tissue specimens from 30 thymoma-associated myasthenia gravis patients. Significantly, the expression levels of miR-20b were inversely correlated with those of both NFAT5 (*r* = −0.629) and CAMTA1 (*r* = −0.589) (Figure 5), indicating that regulation of NFAT5 and CAMTA1 expression by miR-20b occurs not only in cultured cells, but also in patient tissues.

### 3.5. miR-20b Regulates T Cell Proliferation and Activation by NFAT5/CAMTA1

Our previous data demonstrated that miR-20b expression is downregulated in both thymoma and serum samples from patients with thymoma-associated myasthenia gravis.

**Figure 6:**
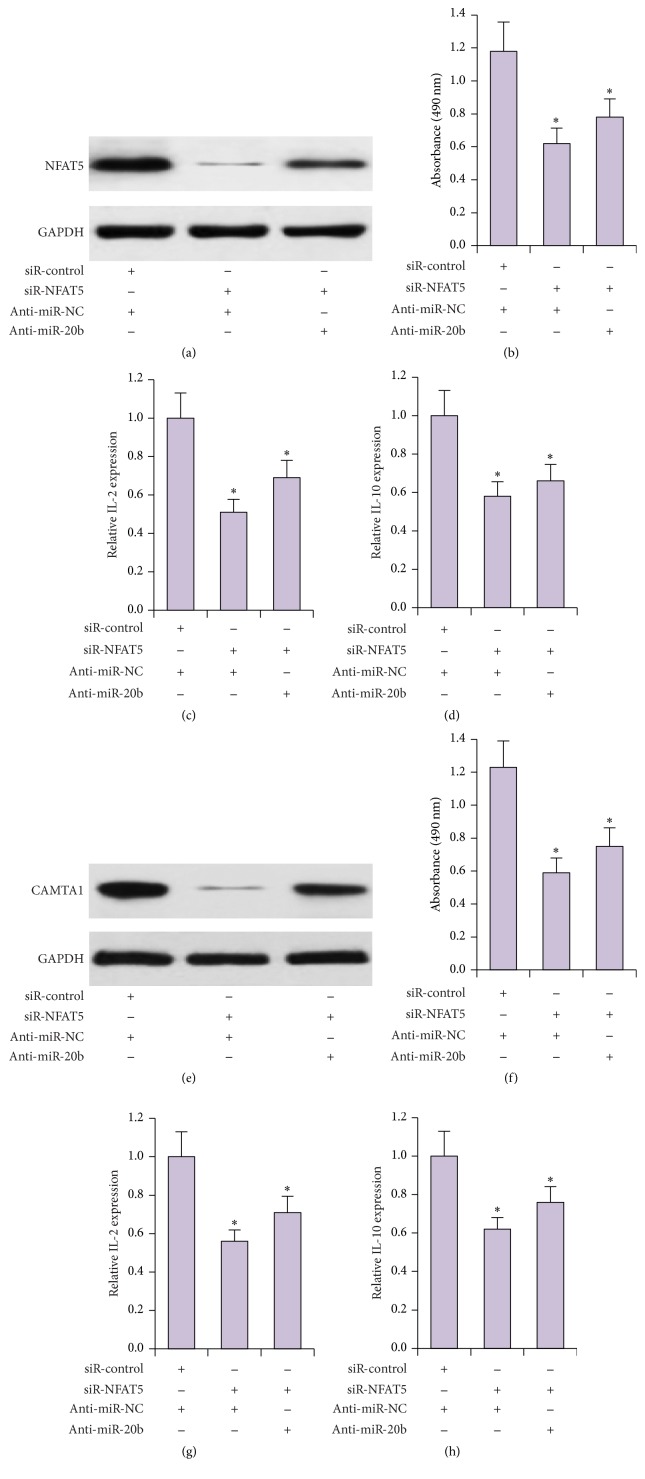
miR-20b affected T cells partially through NFAT5 and CAMTA. (a) NFAT5 expression was determined in siR-control + anti-miR-NC, siR-NFAT5 + anti-miR-NC, and siR-NFAT5 + anti-miR-20b group. MTT cell proliferation (b) and qRT-PCR analysis of IL-2 (c) and IL-10 (d) expression were performed in Jurkat cells. (e) AMTA1 expression was determined in siR-control + anti-miR-NC, siR-CAMTA1 + anti-miR-NC, and siR-CAMTA1 + anti-miR-20b groups. MTT cell proliferation (f) and qRT-PCR analysis of IL-2 (g) and IL-10 (h) expression were performed in Jurkat cells. ^*∗*^ *P* < 0.05.

**Figure 7:**
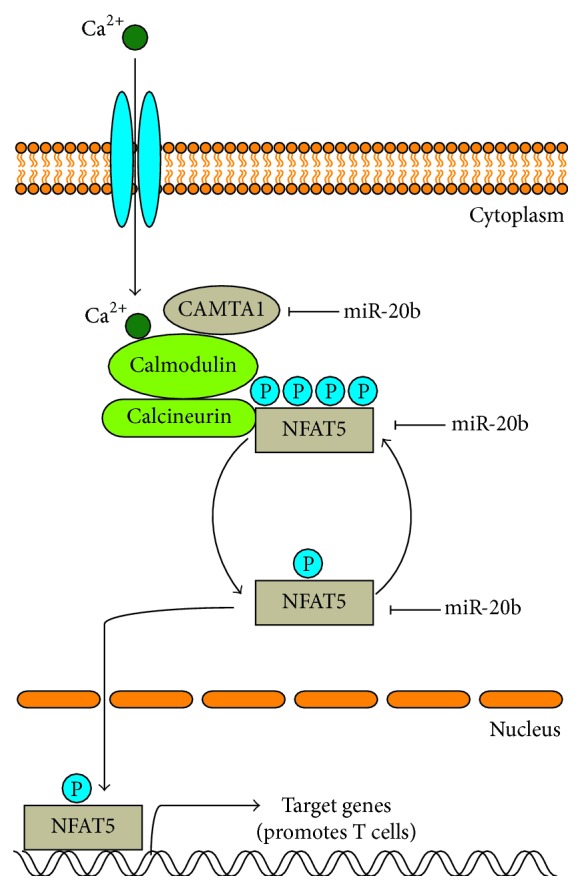
Schematic representation of miR-20b-mediated regulation of the NFAT signaling pathway through repression of NFAT5 and CAMTA1.

miR-20b inhibited T cell proliferation and activation. To verify that miR-20b regulates T cell proliferation and activation through its targets, NFAT5 and CAMTA1, we knocked down NFAT5 and transfected anti-miR-20b into Jurkat cells. We found that NFAT5 expression levels could be partially rescued by anti-miR-20b in Jurkat cells (Figure 6(a)). T cell proliferation and activation assays were performed. As shown in Figures 6(b)–6(d), knockdown of NFAT5 suppressed T cell proliferation and activation. Inhibition of miR-20b partially rescued T cell proliferation and activation affected by NFAT5 downregulation. The results indicate that miR-20b regulates T cell partially through NFAT5. Similarly, we observed that miR-20b controlled T cell proliferation and activation partially through CAMTA1 (Figures 6(e)–6(h)). Taken together, our results suggest that miR-20b regulates T cell proliferation and activation during thymoma development by inhibiting components of the NFAT signaling pathway (Figure 7).

## 4. Discussion

We focused our investigation on the role of miR-20b in thymoma and thymoma-associated myasthenia gravis. We began by examining the levels of miR-20b in thymoma tissue and serum samples from patients with thymoma-associated myasthenia gravis and found that levels of miR-20b were significantly decreased in both. The decreased levels of miR-20b suggested that, in thymoma development, miR-20b likely functions as a tumor suppressor. This result is not consistent with previous studies in other tumor types, in which miR-20b primarily functions as an oncogene or is correlated with poor outcome in liver [14], gastric [13], and lung cancers [17]. Collectively, it would appear that, depending on various targets, miR-20b could act both as oncogene and as tumor suppressor in different tumor types.

Thymomas are a unique type of cancer because they are able to generate mature T cells from immature precursors. We conducted a series of assays to determine the roles of miR-20b in T cell proliferation and found that overexpression of miR-20b inhibited T cell proliferation and activation, while inhibition of miR-20b by anti-miR-20b promoted T cell proliferation and activation. These results were further confirmed by cell cycle analysis, through which we found that miR-20b effectively prevented progression of the cell cycle. In addition, not only was T cell proliferation inhibited, but also miR-20b inhibited the activation of mature T cells. These results together suggested that miR-20b is key in regulating T cell development and can therefore affect thymoma development through this regulation.

We also identified NFAT5 and CAMTA1 as target genes for miR-20b. NFAT5 encodes a transcription factor belonging to a family of proteins that play central roles in regulating gene transcription during the immune response induced by osmotic stress in mammalian cells [23]. NFAT5 is vital to cell cycle progression and proliferation of T cells [24, 25]. Mice without NFAT5 expression suffer from constitutive, pronounced hypernatremia and manifest severe immunodeficiency, with T cell lymphopenia, altered naive/memory T cell homeostasis, and inability to reject allogeneic tumors [26]. CAMTA1 belongs to a protein family that responds to calcium signaling by binding to calmodulin [27]. CAMTA1 has been shown to function as an integrator and effector of calcium signaling and to mediate calcium-dependent processes in neuronal differentiation [28]. A previous study demonstrated that knockdown of CAMTA1 significantly inhibited the activity of NFAT5, possibly by inducing dephosphorylation of NFAT5 [29]. In our study, using a luciferase assay, we were able to confirm the predicted target relationship between miR-20b and NFAT5 and CAMTA1. We also confirmed the inhibitory regulation of NFAT5 and CAMTA1 by miR-20b in cultured cells. Most importantly, we found that, in tissue samples from patients with thymoma-associated myasthenia gravis, the expression levels of miR-20b were correlated inversely with those of NFAT5 and CAMTA1, suggesting that the inhibitory regulation of NFAT5 and CAMTA1 by miR-20b could be implicated in the pathophysiology of thymoma and thymoma-associated myasthenia gravis.
